# Rational Targeting of Cdc42 Overcomes Drug Resistance of Multiple Myeloma

**DOI:** 10.3389/fonc.2019.00958

**Published:** 2019-10-01

**Authors:** Phuong Nguyen, Jayati Chakrabarti, Yuan Li, Khalid W. Kalim, Mengnan Zhang, Lin Zhang, Yi Zheng, Fukun Guo

**Affiliations:** ^1^Division of Experimental Hematology and Cancer Biology, Children's Hospital Medical Center, Cincinnati, OH, United States; ^2^Department of Pediatrics, University of Cincinnati College of Medicine, Cincinnati, OH, United States; ^3^Huiqiao Medical Center, Nanfang Hospital, Southern Medical University, Guangzhou, China; ^4^Key Laboratory of Construction and Detection in Tissue Engineering of Guangdong Province, Department of Histology and Embryology, School of Basic Medical Sciences, Southern Medical University, Guangzhou, China

**Keywords:** multiple myeloma, Cdc42, CASIN, drug resistance, p-ERK

## Abstract

Multiple myeloma (MM) drug resistance highlights a need for alternative therapeutic strategies. In this study, we show that CASIN, a selective inhibitor of cell division cycle 42 (Cdc42) GTPase, inhibited proliferation and survival of melphalan/bortezomib-resistant MM cells more profoundly than that of the sensitive cells. Furthermore, CASIN was more potent than melphalan/bortezomib in inhibiting melphalan/bortezomib-resistant cells. In addition, CASIN sensitized melphalan/bortezomib-resistant cells to this drug combination. Mechanistically, Cdc42 activity was higher in melphalan/bortezomib-resistant cells than that in the sensitive cells. CASIN inhibited mono-ubiquitination of Fanconi anemia (FA) complementation group D2 (FANCD2) of the FA DNA damage repair pathway in melphalan-resistant but not melphalan-sensitive cells, thereby sensitizing melphalan-resistant cells to DNA damage. CASIN suppressed epidermal growth factor receptor (EGFR), signal transducer and activator of transcription 3 (STAT3), and extracellular signal-regulated kinase (ERK) activities to a larger extent in bortezomib-resistant than in melphalan-sensitive cells. Reconstitution of ERK activity partially protected CASIN-treated bortezomib-resistant cells from death, suggesting that CASIN-induced killing is attributable to suppression of ERK. Importantly, CASIN extended the lifespan of mouse xenografts of bortezomib-resistant cells and caused apoptosis of myeloma cells from bortezomib-resistant MM patients. Finally, CASIN had negligible side effects on peripheral blood mononuclear cells (PBMC) from healthy human subjects and normal B cells. Our data provide a proof of concept demonstration that rational targeting of Cdc42 represents a promising approach to overcome MM drug resistance.

## Introduction

Multiple myeloma (MM) is an incurable blood cancer of plasma cells and is the second most prevalent hematological malignancy (1, 2). Melphalan, a DNA cross-linking agent, was the most widely used drug to treat MM patients in the 1960s. However, despite initial responses, most patients develop acquired resistance to melphalan (3). Bortezomib, the first proteasome inhibitor, was introduced into clinical practice for the treatment of relapsed MM (4). Although bortezomib shows an impressive anti-myeloma activity with an overall response rate of 43% (5), patient resistance to this drug has developed (6–8). A second generation of proteasome inhibitors as well as proteasome inhibitors in combination with immunomodulatory drugs (IMIDs), monoclonal antibodies (MoAbs), or histone deacetylase (HDAC) inhibitors have proven effective in overcoming bortezomib resistance (9). Nonetheless, novel therapeutics are desired to further conquer primary or acquired drug resistance of MM.

Numerous signaling pathways are involved in drug resistance of MM. For instance, increased activity of extracellular signal-regulated kinase 1 and 2 (ERK1/2) of the mitogen-activated protein kinase (MAPK) pathway plays a critical role in thioredoxin-mediated bortezomib resistance (10). MAPK kinase (MEK)/ERK and Janus kinase (JAK)/signal transducer and activator transcription 3 (STAT3) pathways have been reported to mediate CDC28 protein kinase regulatory subunit 1B (CKS1B)-induced drug resistance in aggressive CKS1B-overexpressing MM (11). Epidermal growth factor receptor (EGFR)/JAK1/STAT3 signaling is associated with sensitivity of MM cells to proteasome inhibitors (12). In addition, enhanced expression of the Fanconi Anemia (FA) DNA damage repair pathway and elevated mono-ubiquitination of the associated FA complementation group D2 (FANCD2) are critical mechanisms of melphalan resistance (13, 14). Despite these advances in knowledge, the mechanisms underlying MM drug resistance have not been fully elucidated.

The Rho family of small GTPases are a class of molecular switches that cycle between GTP-bound active and GDP-bound inactive forms (15–17). Our previous study showed that B cell-specific deletion of cell division cycle 42 (Cdc42) of Rho GTPases reduces plasma cells (18). Therefore, we question whether Cdc42 could be targeted to benefit patients with MM. In this study, we found that a selective Cdc42 inhibitor, CASIN (19), significantly inhibited proliferation and survival of melphalan- and bortezomib-sensitive and -resistant MM cells. Importantly, CASIN had a stronger effect on melphalan/bortezomib-resistant cells than it did on -sensitive cells. Furthermore, CASIN sensitized melphalan/bortezomib-resistant MM cells to melphalan/bortezomib. Concomitantly, CASIN extended the lifespan of mouse xenografts of bortezomib-resistant MM cells and caused apoptosis of primary myeloma cells from bortezomib-resistant MM patients.

## Materials and Methods

### Reagents

Antibodies against Cdc42 (Cat# 2466S), phosphorylated-EGFR (p-EGFR, Cat# 2234), EGFR (Cat# 2232), p-ERK (Cat# 9102S), ERK (9102), p-AKT (S473, Cat# 4960), p-AKT (T308, Cat# 4056), AKT (Cat# 9272S), and STAT3 (Cat# 4904T) were obtained from Cell Signaling Technology. Antibodies against p-STAT3 (pS727, Cat# 612542) were purchased from BD Biosciences. Anti-Rac1 was obtained from Millipore (Cat# 05-389). Anti-FANCD2 was purchased from Novus Biologicals (Cat# NB100-361). Anti-β-actin antibody was obtained from Santa Cruz (Cat# sc-47778). MEK1 adenovirus was kindly provided by Dr. Jeffery Molkentin (Children'Hospital Medical Center, Cincinnati).

Bortezomib (Cat# S1013) and BVD523 (Cat# 869886-67-9) were obtained from Selleckchem. CASIN was obtained from Cayman Chemical Co. (Cat# 425399-05-9). For the *in vitro* experiments, CASIN was dissolved in DMSO to make the stock solution, followed by diluting it with the culture medium to a series of the testing solutions. For the *in vivo* experiments, CASIN was dissolved in cyclodextran. Melphalan was purchased from Sigma-Aldrich (Cat# 148-82-3). The protease inhibitor cocktail tablets were obtained from Roche Diagnostics GmbH (Ref# 11836170001). The phosphatase inhibitor cocktail was purchased from Goldbio (Cat# GB-450).

### Cell Lines and Culture

The melphalan-resistant RPMI-8226/LR5 (LR5) and melphalan-sensitive RPMI 8226/S (S) MM cell lines were provided by Dr. William S. Dalton and cultured in RPMI1640 medium containing 10% fetal bovine serum (FBS), in the presence or absence of melphalan, as described previously (14). The bortezomib-resistant interleukin (IL)-6-independent RPMI-8226/V10R (V10R) and IL-6-dependent ANBL-6/V10R, and bortezomib-sensitive RPMI-8226/WT (WT) and ANBL-6/WT MM cell lines were provided by Dr. Robert Orlowski and cultured in RPMI1640 medium containing 10% FBS with or without bortezomib or IL-6, as described previously (20–22). EBV-transformed human B cells were provided by Dr. Theodosia Kalfa and were cultured in RPMI1640 medium containing 20% FBS.

### Establishment of Cdc42 Knockdown MM Cells

To generate Cdc42 knockdown MM cells, lentiviral particles containing short hairpin RNA (shRNA) for Cdc42 (Cdc42 shRNA: CCGGCCCTCTACTATTGAGAAACTTCTCGAGAAGTT TCTCAATAGTAGAGGGTTTTTG) or non-targeting shRNA (Scramble shRNA- CCGGGC GCGATAGCGCTAATAATTTCTCGAGAAATTATTAGCGCTATCGCGCTTTTT) were transduced into S and LR5 cells for 8 h. Forty hours later, the cells were flow-sorted for YFP^+^ cells.

### Western Blot

Cells were extracted using radioimmunoprecipitation assay (RIPA) lysis buffer (1× phosphate-buffered saline [PBS], 1% Nonidet P-40, 0.5% sodium deoxycholate, 0.1% sodium dodecyl sulfate [SDS], 1 mM phenyl methyl sulfonyl fluoride, and protease and phosphatase inhibitors). Total cell lysates were centrifuged at 10,000 *g* for 10 min to remove the cell debris, and proteins in the supernatant were fractionated using SDS-polyacrylamide gel electrophoresis, electrophoretically transferred onto polyvinylidene fluoride (PVDF) membrane (Bio-Rad), and probed with the indicated antibodies. The bands were visualized using an enhanced chemiluminescence system (Thermo Scientific).

### Cell Proliferation

After exposing cells to the indicated chemicals for the specified time, viable cells were measured using the 3-(4,5-dimethylthiazol-2-yl)-5-(3-carboxymethoxyphenyl)-2-(4-sulfophenyl)-2H-tetrazolium (MTS) assay following the manufacture's protocol (proliferation assay kit, Promega, CAS# G3580). Briefly, the cells were incubated for 2 h with the kit reagents and then the absorbance at 490 nm was assayed using a microtiter plate reader (Berthold Tech.).

### Flow Cytometry

For cell apoptosis assay, cells were incubated with Annexin V for 15 min, according to the manufacture's protocol (Invitrogen). For DNA damage assay, cells were fixed, permeabilized, and then incubated with anti-γ H2A histone family member X-positive (γH2AX, eBioscience; Cat# 50-9865-42) for 30 min. The cells were then analyzed by flow cytometry.

### Human Samples

Peripheral blood mononuclear cells (PBMC) from healthy human subjects were collected after obtaining informed consent according to the protocol approved by the review board of Cincinnati Children's Hospital Medical Center. The cells were cultured in RPMI1640 medium containing 10% FBS.

PBMC from bortezomib-resistant MM patients were collected by Dr. Stephen Medlin, after obtaining informed consent according to the protocol approved by the review board of University of Cincinnati Medical Center. MM patients with other cancer(s) were excluded. CD138^+^ plasma cells were isolated using magnetic-activated cell sorting. The cells were cultured in RPMI1640 medium containing 10% of FBS and 2 ng/mL recombinant IL-6 (R&D Systems) (23).

### Pull-Down Assay

The activities of Cdc42 (Cdc42-GTP) and Rac1 GTPase (Rac1-GTP) were detected using a previously described pull-down assay, using the Cdc42/Rac1 effector probe GST-PAK1 containing the Cdc42/Rac1-interactive domain (24).

### Xenograft Mouse Model

NSG mice (8–10-week-old) were obtained from Cincinnati Children's hospital. The mice were conditioned by administering busulfan (3 mg/kg) followed by intrafemoral injections of 1 × 10^6^ V10R cells per mouse the next day. Two days after cell injection, the mice were randomly allocated to the following four groups and treated as indicated: vehicle [150 μL/mouse, intraperitoneally (ip)]; CASIN (20 mg/kg, 2 times/day, ip); bortezomib (0.2 mg/kg, 2 times a week, ip); or CASIN plus bortezomib (CASIN, 20 mg/kg, 2 times/day). The mice were monitored daily and euthanized when they developed signs of reduced mobility including paralysis, hunched posture, respiratory distress, or a combination of these signs according to the institutional ethical guidelines. No blinding was performed. All the mice were housed under specific pathogen-free conditions in the animal facility at Cincinnati Children's Hospital Research Foundation. Mice were anesthetized when necessary, using ketamine [80–100 mg/kg, intramuscularly (im)], acepromazine (4–6 mg/kg, im), and atropine (0.1 mg/kg, im). Anesthesia was maintained using ketamine (30 mg/kg, im) as needed. During the experiments, mice were isolated in microisolator cages and cared for in the Laboratory Animal Resource Center by a trained technician and two veterinarians. Animals were checked daily by qualified personnel in the laboratory. The method of euthanasia was CO_2_-induced. The animal study was in compliance with the Cincinnati Children's Hospital Medical Center Animal Care and Use Committee protocols and the National Institutes of Health (NIH) guide for the care and use of Laboratory animals (NIH Publications No. 8023, revised 1978).

### Statistics

For cell line culture experiments, the data represent means ± standard deviation (SD, error bars) of technical replicates (triplicates) from three independent experiments. The results of the MM patient cell culture experiments represent means ± SD of biological replicates (*n* = 4). A two-sided *t*-test was used to determine differences between two experimental groups. For animal experiments, the data represent means ± SD of biological replicates. Each experimental group (Vehicle, CASIN, bortezomib, CASIN plus bortezomib) consisted of 10 mice to ensure a power of 85%, effect size of 1.0, 30% SD, and *P* < 0.05. A log-rank (Mantel-Cox) test was used to determine differences between two experimental groups. For all of the experiments, *P* < 0.05 or *P* < 0.01 was considered statistically significant.

## Results

### Cdc42 Is Essential for MM Cell Proliferation and Survival

**Figure 1:**
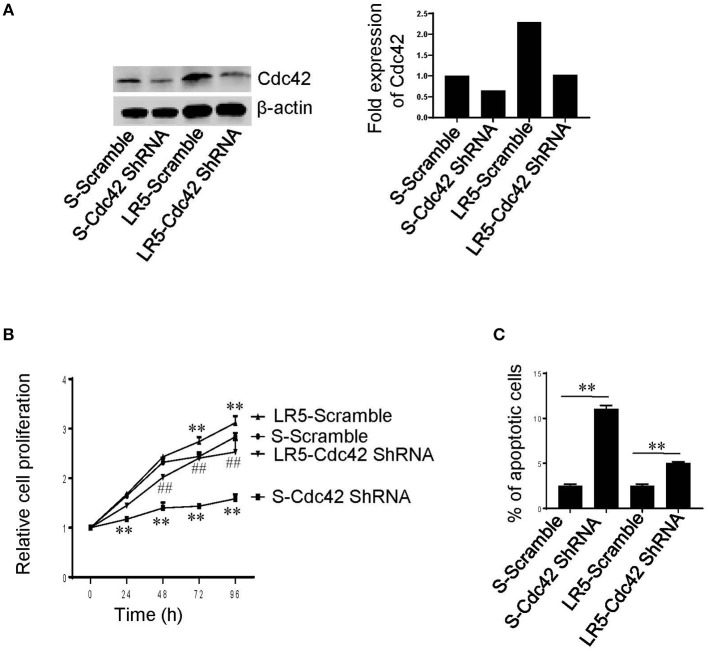
Cdc42 knockdown inhibits proliferation and causes apoptosis of MM cells. **(A)** Western blot analysis of Cdc42, following transduction of melphalan-sensitive (S) and melphalan-resistant (LR5) MM cells with Scramble shRNA (Scramble) or Cdc42 shRNA. β-actin was used as loading control (left). The expression levels of Cdc42 were quantified (right). **(B)** Proliferation of S and LR5 cells. **P < 0.01 vs. S-Scramble; ^##^*P* < 0.01 vs. LR5-Scramble. **(C)** Apoptosis of S and LR5 cells. Annexin V^+^ cells were analyzed using flow cytometry. **P < 0.01. Error bars represent means ± SD of triplicates and data are representative of three independent experiments.

To determine whether Cdc42 has a role in MM cell proliferation and survival, we used Cdc42 shRNA to knockdown Cdc42 in S and LR5 cells (Figure 1A). We found that Cdc42 knockdown significantly inhibited cell proliferation (Figure 1B) and caused cell apoptosis (Figure 1C) in both S and LR5 cells, suggesting that Cdc42 plays an important role in MM cell proliferation and survival.

### Half-Maximal Growth Inhibition Concentration of CASIN

**Figure 2:**
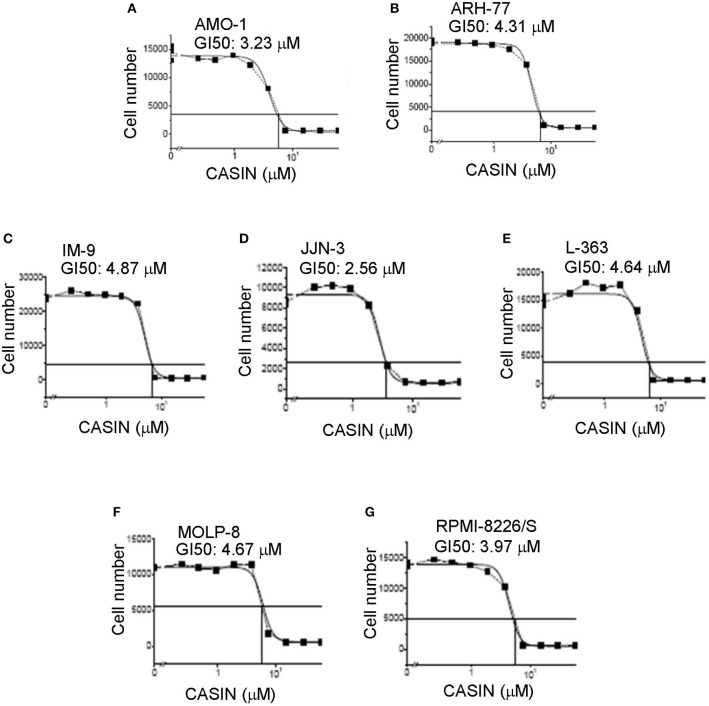
Half-maximal growth inhibitory concentration (GI50) of CASIN ranges from 2.56 to 4.87 μM in different MM cell lines. **(A)** GI50 of AMO-1 cells. **(B)** GI50 of ARH-77 cells. **(C)** GI50 of IM-9 cells. **(D)** GI50 of JJN-3 cells. **(E)** GI50 of L-363 cells. **(F)** GI50 of MOLP-8 cells. **(G)** GI50 of RPMI-8226/S cells. Indicated cell lines were plated at 2,000 cells/well and treated with or without different concentrations of CASIN for 3 days. Cell numbers were then counted. Data are representative of three independent experiments.

Considering the role of Cdc42 in MM cell proliferation and survival, we postulated that targeting of Cdc42 might benefit MM patients. To test this hypothesis, we examined the effects of a Cdc42 inhibitor, CASIN (19). We first determined the half-maximal growth inhibition concentration (GI_50_) of CASIN in a number of MM cell lines and found that it ranged from 2.56 to 4.87 μM (Figures 2A–G). CASIN was used at 5 μM, unless otherwise noted, in all subsequent *in vitro* studies.

### CASIN Preferentially Suppresses Melphalan-Resistant MM Cells

**Figure 3:**
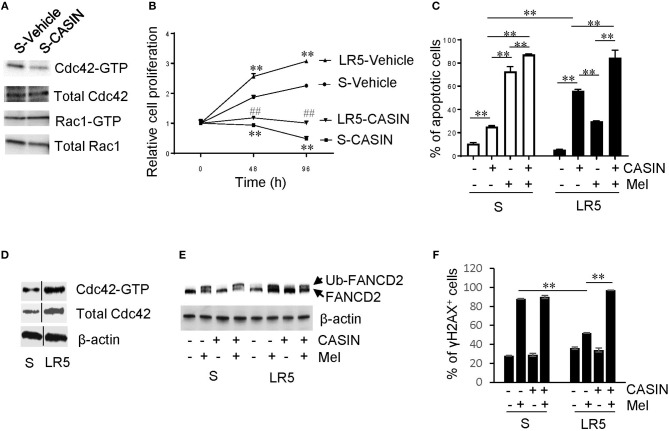
Effects and mechanism of action of CASIN on melphalan-resistant MM cells. **(A)** CASIN reduces Cdc42 activity (Cdc42-GTP) but not Rac1 activity (Rac1-GTP). Melphalan-sensitive MM cells (S) were treated with Vehicle or CASIN (5 μM) for 8 h. Cdc42 and Rac1 activities were measured using pull-down assay. **(B)** CASIN (5 μM) inhibits proliferation of both S and melphalan-resistant (LR5) MM cells. **P < 0.01 vs. S-Vehicle; ^##^*P* < 0.01 vs. LR5-Vehicle. **(C)** CASIN (5 μM) preferentially causes apoptosis of melphalan-resistant MM cells. S and LR5 cells were treated with or without CASIN or melphalan (Mel, 25 μM) or both for 2 days. Annexin V^+^ cells were analyzed using flow cytometry. **P < 0.01. **(D–F)** Mechanism of action of CASIN on melphalan-resistant MM cells. **(D)** Cdc42 activity and expression are increased in LR5 cells compared to that in S cells. Cdc42 activity was measured using pull-down assay. β-Actin was used as loading control. Vertical lines indicate the gel lanes being switched in position from the original blots. **(E)** CASIN (5 μM) decreases melphalan-induced FANCD2 mono-ubiquitination (Ub-FANCD2) in LR5 but not in S cells. S and LR5 cells were treated with or without CASIN, Mel (25 μM), or both for 16 h. FANCD2 was detected using western blotting. β-Actin was used as loading control. **(F)** CASIN sensitizes melphalan-resistant but not -sensitive MM cells to melphalan-induced DNA damage. γ H2A histone family member X-positive (γH2AX^+^) cells were detected using flow cytometry. **P < 0.01. Error bars represent means ± SD of triplicates and data are representative of three independent experiments.

We used S cells to confirm that CASIN selectively inhibited Cdc42 activity without affecting that of Rac1, another Rho GTPase family member closely related to Cdc42 (Figure 3A). We found that CASIN suppressed the proliferation of S and LR5 cells to a similar extent (Figure 3B), with a GI50 of 5.454 μM for LR5 cells (Supplemental Figure S1A). CASIN also caused apoptosis of both S and LR5 cells (Figure 3C). Notably, CASIN was more potent in inducing apoptosis of LR5 cells than that of S cells (10- and 2.5-fold increase, respectively, compared to vehicle group, Figure 3C). While CASIN was less effective than melphalan in killing S cells, it was more effective than melphalan in killing LR5 cells (Figure 3C). Moreover, CASIN sensitized both S and LR5 cells to melphalan-induced cell death. Intriguingly, the sensitizing effect of CASIN was more profound in LR5 than in S cells (Figure 3C). Our data indicate that CASIN could be particularly effective in counteracting melphalan resistance in MM.

### Mechanisms of Action of CASIN in Suppressing Melphalan-Resistant MM Cells

Next, we wanted to understand the mechanisms underlying the dominant effect of CASIN on melphalan-resistant cells over their sensitive counterparts, and we detected an increase in Cdc42 protein expression and activity in LR5 cells (Figures 1A, 3D). Furthermore, we wondered why CASIN was more effective in sensitizing melphalan-resistant cells to melphalan-induced cell death. It has been shown that melphalan resistance is attributable to increased mono-ubiquitination of FANCD2 of the FA DNA damage repair pathway and its mediated DNA repair (13). Consistently, we found that the level of melphalan-induced FANCD2 mono-ubiquitination was higher and DNA damage was lower in LR5 cells than that in S cells, as evidenced by decreased frequency of γH2AX^+^ cells (Figures 3E,F). Interestingly, CASIN abolished melphalan-induced FANCD2 mono-ubiquitination in LR5 but not S cells (Figure 3E). Concomitantly, CASIN increased melphalan-induced DNA damage in LR5 but not S cells, as evidenced by increased frequency of γH2AX^+^ cells (Figure 3F). Our data indicate that CASIN sensitizes melphalan-resistant cells to melphalan-induced cell death by blocking FANCD2-mediated DNA damage repair.

### CASIN Preferentially Suppresses Bortezomib-Resistant MM Cells

**Figure 4:**
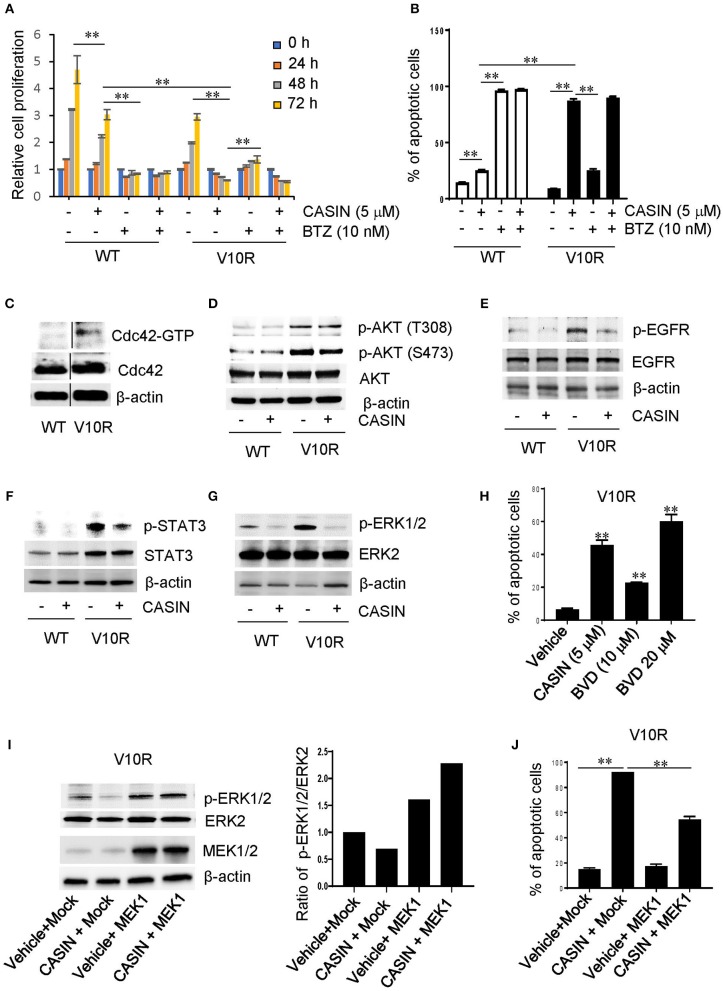
Effects and mechanism of action of CASIN in bortezomib-resistant MM cells. **(A)** CASIN preferentially suppresses cell proliferation in bortezomib-resistant MM cells. Bortezomib-sensitive MM cells (WT) and bortezomib-resistant MM cells (V10R) were treated with or without CASIN (5 μM), bortezomib (BTZ) (10 nM) for the indicated time. Cell proliferation was then measured. **P < 0.01 (comparisons were made for 48 and 72 h). **(B)** CASIN preferentially causes cell apoptosis in bortezomib-resistant MM cells. WT and V10R cells were treated with or without CASIN (5 μM), BTZ (10 nM), or both for 2 days. Cell apoptosis was determined using flow cytometry analysis of Annexin V^+^ cells. **P < 0.01. **(C–J)** Mechanism of action of CASIN in bortezomib-resistant MM cells. **(C)** Cdc42 activity is increased in bortezomib-resistant MM cells. Cdc42 activity in WT and V10R cells was measured using pull-down assay. β-Actin was used as loading control. Vertical lines indicate the gel lanes being switched in position from the original blots. **(D)** CASIN has no effect on AKT activity in bortezomib-resistant MM cells. WT and V10R cells were treated with or without CASIN (5 μM) for 8 h. p-AKT and total AKT were detected using western blot analysis. β-Actin was used as loading control. **(E–G)** CASIN suppresses epidermal growth factor receptor (EGFR), signal transducer and activator of transcription 3 (STAT3), and extracellular signal-regulated kinase (ERK) activities in bortezomib-resistant MM cells. WT and V10R cells were treated with or without CASIN for 8 h. p-EGFR and total EGFR **(E)**, STAT3 **(F)**, and ERK1/2 **(G)** were detected using western blot analysis. β-Actin was used as loading control. **(H)** Inhibition of ERK mimics CASIN in inducing cell apoptosis. V10R cells were treated with or without CASIN (5 μM) or ERK inhibitor BVD532 (BVD, 10 or 20 μM) for 2 days. Cell apoptosis was determined using flow cytometry of Annexin V^+^ cells. **P < 0.01 vs. Vehicle. **(I,J)** Restoration of ERK activity in bortezomib-resistant MM cells partially rescues apoptosis of bortezomib-resistant MM cells. V10R cells were transduced with adenoviral mitogen-activated protein kinase 1 (MEK1) or Mock. The cells were then treated with CASIN or vehicle for 8 h. p-ERK1/2, total ERK2, and MEK1 were detected using western blot analysis (**I**—left). β-actin was used as loading control (**I**—left). The ratio of p-ERK1/2 vs. total ERK2 was quantified (**I**—right). For examination of cell apoptosis, cells were treated with CASIN or vehicle for 2 days and then analyzed for Annexin V^+^ cells using flow cytometry **(J)**. **P < 0.01. Error bars represent means ± SD of triplicates and data are representative of three independent experiments.

We then determined whether CASIN could ameliorate bortezomib resistance. CASIN (5 μM) inhibited the proliferation (Figure 4A) and survival (Figure 4B) of bortezomib-resistant V10R cells to a larger extent than -sensitive WT cells, with a GI50 of 4.191 μM for V10R cells (Supplemental Figure S1B). While CASIN was less effective than bortezomib (10 nM) in suppressing WT cells, it was substantially more potent in inhibiting V10R cells (Figures 4A,B). Treatment of V10R cells with CASIN at a lower concentration (3 μM) increased cell sensitivity to bortezomib (7.5 and 10 nM, Supplemental Figures S2A,B). Similarly, CASIN more profoundly inhibited the IL-6-dependent bortezomib-resistant MM cells, ANBL-6/V10R (Supplemental Figures S3A,B). CASIN also increased bortezomib sensitivity of ANBL-6/V10R cells (Supplemental Figures S3A,B).

### Mechanism of Action of CASIN in Suppressing Bortezomib-Resistant MM Cells

Similar to melphalan-resistant MM cells, bortezomib-resistant V10R cells showed elevated Cdc42 activity (Figure 4C). Thus, increased Cdc42 activity seemed to contribute to bortezomib resistance and the superior efficiency of CASIN in suppressing bortezomib-resistant MM cells. Recent studies have shown that targeting of PI-3 kinase/AKT signaling pathway enhances the sensitivity of MM cells to bortezomib and overcomes bortezomib resistance (25, 26). We confirmed that the activity of AKT was markedly elevated in bortezomib-resistant V10R cells compared to that in the sensitive WT cells (Figure 4D). However, CASIN did not affect the activity of AKT in V10R cells (Figure 4D), suggesting that PI-3 kinase/AKT pathway does not contribute to the effects of CASIN on bortezomib-resistant MM cells. EGFR/STAT3 signaling pathway is also involved in the sensitivity of MM cells to bortezomib (12). Consistently, both p-EGFR and p-STAT3 were elevated in V10R cells (Figures 4E,F). The increased activities of EGFR and STAT3 were suppressed by CASIN treatment, suggesting that the dominant effects of CASIN on bortezomib-resistant MM cell proliferation and survival are attributable to its suppression of EGFR/STAT3 signaling pathway. In addition, ERK signaling is associated with chemo-resistance of MM (10, 12). We found that the activity of ERK, which was increased in V10R cells (Figure 4G), was attenuated by CASIN (Figure 4G). Treatment of V10R cells with an ERK inhibitor, BVD523, mimicked the effect of CASIN on inhibition of V10R cell survival (Figure 4H). Importantly, restoration of ERK activity in CASIN-treated V10R cells by viral transduction of MEK1 (Figure 4I) partially inhibited the apoptosis of V10R cells (Figure 4J), demonstrating that inhibition of ERK pathway critically contributed to the inhibitory effect of CASIN on survival of bortezomib-resistant cells. Collectively, these results imply that the mechanism of action of CASIN on bortezomib-resistant MM cells involved inhibition of EGFR/STAT3 and ERK signaling pathways.

### CASIN Prolongs Lifespan of Mouse Xenografts of Bortezomib-Resistant MM Cells and Causes Apoptosis of Bortezomib-Resistant MM Patient Cells

**Figure 5:**
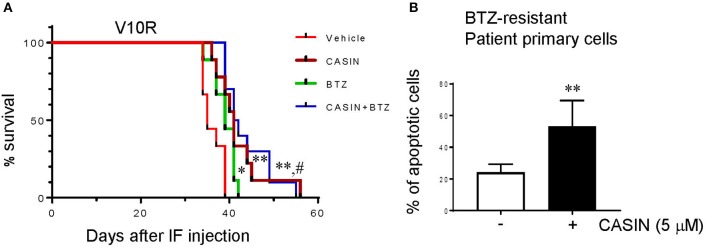
**(A)** CASIN prolongs lifespan of mice bearing bortezomib-resistant MM cells. NSG mice were subjected to myeloablative busulfan conditioning followed by intrafemoral injection of V10R cells. The mice were then treated with Vehicle, CASIN, bortezomib (BTZ), or CASIN + BTZ. Mouse survival was recorded. Data were analyzed using log-rank (Mantel-Cox) test, *n* = 10 mice. *P <0.05 vs. Vehicle, **P < 0.01 vs. Vehicle and ^#^*P* < 0.01 vs. BTZ. **(B)** CASIN causes apoptosis of primary cells from bortezomib-resistant MM patients. Human CD138^+^ cells were isolated from peripheral blood of bortezomib (BTZ)-resistant MM patients. The cells were treated with or without CASIN (5 μM) for 2 days. Cell apoptosis was determined using flow cytometry analysis of Annexin V^+^ cells. Error bars represent means ± SD of four patients, **P < 0.01.

Considering the potency of CASIN in bortezomib-resistant MM cells *in vitro*, we examined its efficacy in mouse xenografts of bortezomib-resistant cells. As shown in Figure 5A, while bortezomib treatment only slightly extended the lifespan of V10R cell-bearing mice, the CASIN- and combined CASIN and bortezomib-treated V10R cell-bearing mice exhibited a significantly prolonged lifespan. Of note, the lifespan of the co-treated group was comparable to that of the CASIN group, suggesting that the effect of the co-treatment was mostly mediated by CASIN, similar to the *in vitro* effect of co-treatment (Figures 4A,B). Perhaps, a lower dose of CASIN would sensitize V10R-xenografted mice to bortezomib, as seen *in vitro* (Supplemental Figure S2). Our data indicate that CASIN may benefit bortezomib-resistant MM patients. To substantiate this notion, we tested the effect of CASIN on human CD138^+^ primary myeloma cells from bortezomib-resistant MM patients. As shown in Figure 5B, CASIN treatment increased apoptosis of MM patient cells by more than 2-fold. Taken together, these results suggest that Cdc42 could serve as a potential therapeutic target to overcome drug resistance of MM.

### CASIN Has Negligible Side Effects

**Figure 6:**
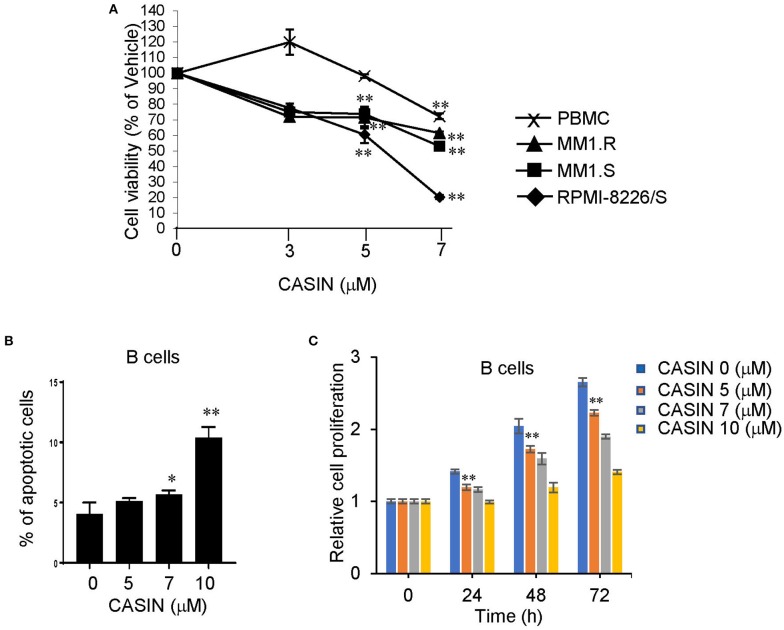
CASIN has negligible side effects. **(A)** CASIN (5 μM) inhibits viability of MM cell lines but not healthy human peripheral blood mononuclear cells (PBMC). RPMI-8226/S, MM1.S, and MM1.R MM cells and PBMC were treated with or without the indicated concentrations of CASIN for 4 days. Viable cells were measured using 3-(4,5-dimethylthiazol-2-yl)-5-(3-carboxymethoxyphenyl)-2-(4-sulfophenyl)-2H-tetrazolium (MTS) assay. Data were normalized to Vehicle group (0 μM CASIN), **P < 0.01 vs. Vehicle group. **(B)** CASIN (5 μM) does not cause apoptosis of human B cells. Human B cells were treated with or without the indicated concentrations of CASIN for 2 days. Cell apoptosis was determined using flow cytometry analysis of Annexin V^+^ cells; *P < 0.05 and **P < 0.01 vs. Vehicle group. **(C)** CASIN (5 μM) has modest inhibitory effect on proliferation of human B cells. Human B cells were treated with or without the indicated concentrations of CASIN for the indicated time. Cell proliferation was then measured. **P < 0.01 vs. Vehicle group at same time point. Error bars represent means ± SD of triplicates and data are representative of three independent experiments.

Finally, we determined the potential side effects of CASIN. We found that 7 μM of CASIN inhibited the viability of both PBMC from healthy human subjects and MM cells (Figure 6A). While CASIN at 5 μM used for all the *in vitro* experiments significantly reduced the viability of MM cells, it did not inhibit PBMC (Figure 6A). CASIN at 5 μM also had no effect on the survival of normal B cells (Figure 6B), and only modestly suppressed their proliferation (Figure 6C). Furthermore, we did not note significant weight loss in the above mouse xenografts treated with CASIN (data not shown). Our data suggest that the side effects of CASIN would be limited.

## Discussion

MM remains an incurable disease and nearly all patients eventually develop resistance to currently available drugs (27). Thus, novel therapeutics that conquer drug resistance of MM are urgently needed. In this study, we provide evidence that CASIN, a Cdc42 inhibitor, of which we own intellectual property (19), represents a promising experimental drug to circumvent melphalan/bortezomib resistance of MM.

We suggest that CASIN overcomes melphalan resistance by decreasing FANCD2 mono-ubiquitination and, subsequently, increasing DNA damage. Considering that bortezomib also inhibits FANCD2 mono-ubiquitination (14), it would be interesting to examine whether bortezomib augments CASIN activity in melphalan-resistant MM cells. On the other hand, CASIN may overcome bortezomib resistance through inhibiting the activities of EGFR, STAT3, and ERK, but not AKT. We have provided evidence to support that CASIN inhibits bortezomib-resistant cell survival partially through inactivating ERK. Whether inactivation of EGFR and STAT3 critically contribute to CASIN-mediated suppression of bortezomib-resistant cell survival needs further investigation. Overexpression of the β5 proteasomal subunit, the primary target of bortezomib, and c-Maf have been shown to regulate bortezomib resistance (28). Whether CASIN suppresses the β5 proteasomal subunit and c-Maf expression remains to be determined. In addition, both melphalan and bortezomib resistance can be conferred by upregulation of multidrug resistance (MDR) proteins (e.g., P-glycoprotein) and glycolytic signaling (28). Thus, examining whether CASIN dampens MDR protein expression and glycolytic signaling is warranted.

An interesting finding of our study is that Cdc42 activity is increased in both melphalan- and bortezomib-resistant MM cells, suggesting that enhanced Cdc42 activity contributes to both melphalan and bortezomib resistance. Thus, our observations that inhibition of Cdc42 activity by CASIN inhibits FANCD2 mono-ubiquitination in melphalan-resistant cells and EGFR, STAT3, and ERK activities in bortezomib-resistant cells suggest that Cdc42 acts upstream of FANCD2 to confer melphalan resistance and upstream of EGFR, STAT3, and ERK to confer bortezomib resistance. An increase in Cdc42 activity in melphalan/bortezomib-resistant cells also indicates that enhanced Cdc42 activity may be used as a biomarker to identify melphalan/bortezomib-resistant MM patients who are most likely to benefit from CASIN treatment.

CASIN acts by binding to inactive GDP-bound Cdc42 to inhibit its cycling to active GTP-bound Cdc42 and is highly specific to Cdc42 (19). To confirm its specificity, we show that 5 μM CASIN does not affect the activity of the closely related Rac1. We believe that CASIN has great applicability for treating drug-resistant MM considering the following points: (1) Cdc42 activity is enhanced in melphalan/bortezomib-resistant MM cells, suggesting that CASIN would likely cause an oncogene addiction-like effect; (2) CASIN only marginally affects healthy human PBMC and human B cells; (3) CASIN has no effect on thymocyte development and T cell homeostasis in mice (29); and (4) CASIN does not cause weight loss and systemic inflammation in steady-state mice and in mice bearing bortezomib-resistant MM cells (data not shown). Thus, a therapeutic window is presumably achievable in clinical treatment of drug-resistant MM patients with CASIN.

In conclusion, our study provides a proof of concept that rational targeting of Cdc42 may be utilized to treat drug-resistant MM and, therefore, warrants clinical evaluation of CASIN in drug-resistant MM patients.

## Data Availability Statement

The raw data supporting the conclusions of this manuscript will be made available by the authors, without undue reservation, to any qualified researcher.

## Ethics Statement

The studies involving human participants were reviewed and approved by Cincinnati Children's Hospital Medical Center. The patients/participants provided their written informed consent to participate in this study. The animal study was reviewed and approved by Cincinnati Children's Hospital Medical Center.

## Author Contributions

PN designed and performed the research, analyzed the data, and wrote the paper. JC and YL performed the research and analyzed the data. KK and MZ performed the research. LZ contributed vital new reagents or analytical tools. YZ designed the research and analyzed the data. FG designed the research, analyzed the data, contributed vital new reagents or analytical tools, and wrote the paper.

### Conflict of Interest

The authors declare that the research was conducted in the absence of any commercial or financial relationships that could be construed as a potential conflict of interest.

## Supplementary Material

Supplemental Figure S1 Half-maximal growth inhibitory concentration (GI50) of CASIN in melphalan- and bortezomib-resistant MM cells. LR5 **(A)** and V10R **(B)** cells were plated at 10,000 cells/well and treated with or without different concentrations of CASIN for 2 days. Viable cells were measured using MTS as described in the Materials and Methods.

Supplemental Figure S2 CASIN (3 μM) sensitizes Bortezomib-resistant MM cells for Bortezomib-induced cell death. **(A)** Bortezomib-resistant MM cells (V10R) were treated with or without the indicated concentrations of CASIN and 10 nM of Bortezomib (BTZ) for 2 days. Cell apoptosis (Annexin V+ cells) was analyzed by flow cytometry. ^**^*P* < 0.01. **(B)** V10R cells were treated with or without the indicated concentrations of BTZ and 3 μM of CASIN for 2 days. Cell apoptosis (Annexin V+ cells) was analyzed by flow cytometry. ^**^*P* < 0.01. Error bars represent mean ± SD of triplicates. Data are representative of three independent experiments.

Supplemental Figure S3 Effects of CASIN on IL-6-dependent Bortezomib-resistant MM cells. **(A)** CASIN preferentially suppresses cell proliferation in IL-6-dependent Bortezomib-resistant ANBL-6/V10R cells. ANBL-6/V10R and IL-6-dependent Bortezomib-sensitive ANBL-6/WT cells were treated with or without CASIN (5 μM) and/or Bortezomib (BTZ) (10 nM) for the indicated time. Cell proliferation was then measured. ^**^*P* < 0.01 (comparisons were made for 72 h). **(B)** CASIN preferentially causes cell apoptosis in IL-6-dependent Bortezomib-resistant ANBL 6/V10R cells. ANBL-6/V10R and ANBL-6/WT cells were treated with or without CASIN (5 μM) and/or BTZ (10 nM) for 2 days. Cell apoptosis was then measured. ^**^*P* < 0.01. Error bars represent mean ± SD of triplicates. Data are representative of three independent experiments.

## References

[1] Collins CD. Problems monitoring response in multiple myeloma. Cancer Imaging. (2005) 5:S119–26. 10.1102/1470-7330.2005.0033 16361127 PMC1665317

[2] Palumbo AM Anderson KM. Multiple myeloma. N Engl J Med. (2011) 364:1046–60. 10.1056/NEJMra1011442 21410373

[3] Bellamy WT Dalton WS Gleason MC Grogan TM Trent JM. Development and characterization of a melphalan-resistant human multiple myeloma cell line. Cancer Res. (1991) 51:995–1002. 1988143

[4] Hideshima T Richardson P Chauhan D Palombella VJ Elliott PJ Adams J. The proteasome inhibitor PS-341 inhibits growth, induces apoptosis, and overcomes drug resistance in human multiple myeloma cells. Cancer Res. (2001) 61:3071–6. 11306489

[5] Richardson PG Sonneveld P Schuster MW Irwin D Stadtmauer EA Facon T Bortezomib or high-dose dexamethasone for relapsed multiple myeloma. N Engl J Med. (2005) 352:2487–98. 10.1056/NEJMoa043445 15958804

[6] McConkey DJ Zhu K. Mechanisms of proteasome inhibitor action and resistance in cancer. Drug Resist Updat. (2008) 11:164–79. 10.1016/j.drup.2008.08.002 18818117

[7] Orlowski RZ Kuhn DJ. Proteasome inhibitors in cancer therapy: lessons from the first decade. Clin Cancer Res. (2008) 14:1649–57. 10.1158/1078-0432.CCR-07-2218 18347166

[8] Niewerth D Jansen G Assaraf YG Zweegman S Kaspers GJ Cloos J. Molecular basis of resistance to proteasome inhibitors in hematological malignancies. Drug Resist Updat. (2015) 18:18–35. 10.1016/j.drup.2014.12.001 25670156

[9] Anderson KC. Progress and paradigms in multiple myeloma. Clin Cancer Res. (2016) 22:5419–27. 10.1158/1078-0432.CCR-16-0625 28151709 PMC5300651

[10] Zheng Z Fan S Zheng J Huang W Gasparetto C Chao NJ. Inhibition of thioredoxin activates mitophagy and overcomes adaptive bortezomib resistance in multiple myeloma. J Hematol Oncol. (2018) 11:29. 10.1186/s13045-018-0575-7 29482577 PMC5828316

[11] Shi L Wang S Zangari M Xu H Cao TM Xu C. Over-expression of CKS1B activates both MEK/ERK and JAK/STAT3 signaling pathways and promotes myeloma cell drug-resistance. Oncotarget. (2010) 1:22–3. 10.18632/oncotarget.105 20930946 PMC2949973

[12] Zhang X-D Baladandayuthapani V Lin H Mulligan G Li B Esseltine D-LW. Tight junction protein 1 modulates proteasome capacity and proteasome inhibitor sensitivity in multiple myeloma via EGFR/JAK1/STAT3 signaling. Cancer Cell. (2016) 29:639–52. 10.1016/j.ccell.2016.03.026 27132469 PMC4983190

[13] Chen Q Van der Sluis PC Boulware D Hazlehurst LA Dalton WS. The FA/BRCA pathway is involved in melphalan-induced DNA interstrand cross-link repair and accounts for melphalan resistance in multiple myeloma cells. Blood. (2005) 106:698–705. 10.1182/blood-2004-11-4286 15802532 PMC1895179

[14] Yarde DN Oliveira V Mathews L Wang X Villagra A Boulware D. Targeting the Fanconi anemia/BRCA pathway circumvents drug resistance in multiple myeloma. Cancer Res. (2009) 69:9367–75. 10.1158/0008-5472.CAN-09-2616 19934314 PMC4519834

[15] Etienne-Manneville S Hall A. Rho GTPases in cell biology. Nature. (2002) 420:629–35. 10.1038/nature01148 12478284

[16] Mulloy JC Cancelas JA Filippi MD Kalfa TA Guo F Zheng Y. Rho GTPases in hematopoiesis and hemopathies. Blood. (2010) 115:936–47. 10.1182/blood-2009-09-198127 19965643 PMC2817638

[17] Melendez J Grogg M Zheng Y. Signaling role of Cdc42 in regulating mammalian physiology. J Biol Chem. (2011) 286:2375–81. 10.1074/jbc.R110.200329 21115489 PMC3024731

[18] Guo F Velu CS Grimes HL Zheng Y. Rho GTPase Cdc42 is essential for B-lymphocyte development and activation. Blood. (2009) 114:2909–16. 10.1182/blood-2009-04-214676 19671922 PMC2756201

[19] Liu W Du W Shang X Wang L Evelyn C Florian MC. Rational identiication of a Cdc42 inhibitor presents a new regimen for long-term hematopoietic stem cell mobilization. Leukemia. (2019) 33:749–61. 10.1038/s41375-018-0251-5 30254339 PMC6414073

[20] Kuhn DJ Chen Q Voorhees PM Strader JS Shenk KD Sun CM. Potent activity of carfilzomib, a novel, irreversible inhibitor of the ubiquitin-proteasome pathway, against preclinical models of multiple myeloma. Blood. (2007) 110:3281–90. 10.1182/blood-2007-01-065888 17591945 PMC2200918

[21] Kuhn DJ Hunsucker SA Chen Q Voorhees PM Orlowski M Orlowski RZ. Targeted inhibition of the immunoproteasome is a potent strategy against models of multiple myeloma that overcomes resistance to conventional drugs and nonspecific proteasome inhibitors. Blood. (2009) 113:4667–76. 10.1182/blood-2008-07-171637 19050304 PMC2680370

[22] Kuhn DJ Berkova Z Jones RJ Woessner R Bjorklund CC Ma W Targeting the insulin-like growth factor-1 receptor to overcome bortezomib resistance in pre-clinical models of multiple myeloma. Blood. (2012) 120:3260–70. 10.1182/blood-2011-10-386789 22932796 PMC3476538

[23] De Smedt E Maes K Verhulst S Lui H Kassambara A Maes A. Loss of RASSF4 expression in multiple myeloma promotes RAS-driven malignant progression. Cancer Res. (2018) 78:1155–68. 10.1158/0008-5472.CAN-17-1544 29259009

[24] Liliental J Moon SY Lesche R Mamillapalli R Li D Zheng Y. Genetic deletion of the Pten tumor suppressor gene promotes cell motility by activation of Rac1 and Cdc42 GTPases. Curr Biol. (2000) 10:401–4. 10.1016/S0960-9822(00)00417-6 10753747

[25] Que W Chen J Chuang M Jiang D. Knockdown of c-Met enhances sensitivity to bortezomib in human multiple myeloma U266 cells via inhibiting Akt/mTOR activity. APMIS. (2012) 120:195–203. 10.1111/j.1600-0463.2011.02836.x 22339676

[26] Yu W Chen Y Xiang R Xu W Wang Y Tong J. Novel phosphatidylinositol 3-kinase inhibitor BKM120 enhances the sensitivity of multiple myeloma to bortezomib and overcomes resistance. Leuk Lymphoma. (2017) 58:428–37. 10.1080/10428194.2016.1190968 27439454

[27] Abdi J Chen G Chang H. Drug resistance in multiple myeloma: latest findings and new concepts on molecular mechanisms. Oncotarget. (2013) 4:2186–207. 10.18632/oncotarget.1497 24327604 PMC3926819

[28] Robak P Drozdz I Szemraj J Robak T. Drug resistance in multiple myeloma. Cancer Treat Rev. (2018) 70:199–208. 10.1016/j.ctrv.2018.09.001 30245231

[29] Yang JQ Kalim KW Li Y Duan X Nguyen P Khurana Hershey GK. Rational targeting Cdc42 restrains Th2 cell differentiation and prevents allergic airway inflammation. Clin Exp Allergy. (2019) 49:92–107. 10.1111/cea.13293 30307073 PMC6310654

