# Supplementary figures and images for: Rational Targeting of Cdc42 Overcomes Drug Resistance of Multiple Myeloma
Source: Front Oncol. 2019 Oct 1;9:958. doi: 10.3389/fonc.2019.00958 (PMC6779689; doi:10.3389/fonc.2019.00958)

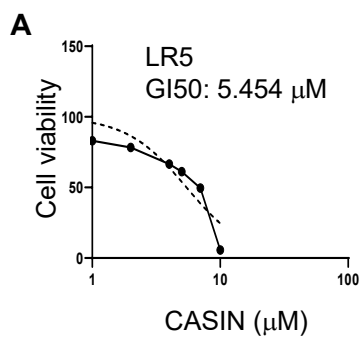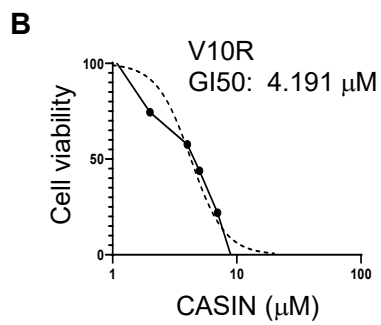

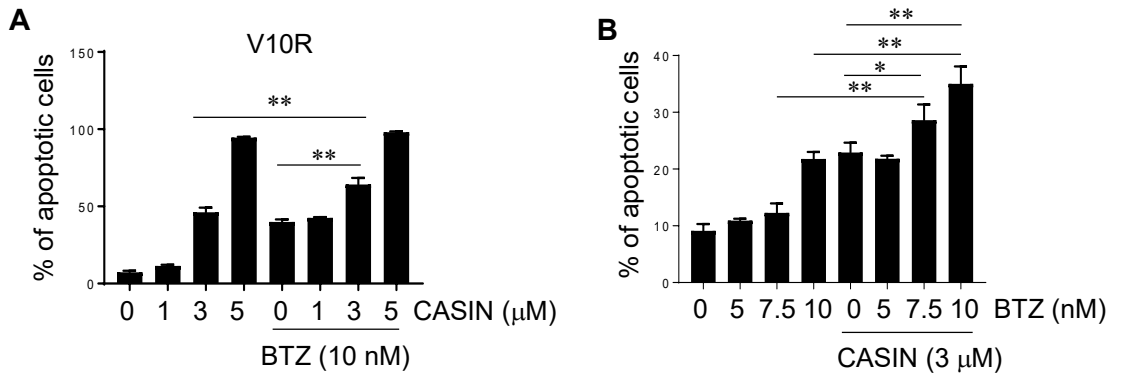

**A**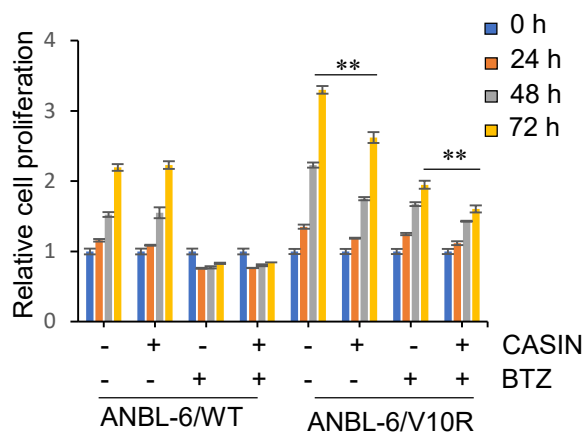**B**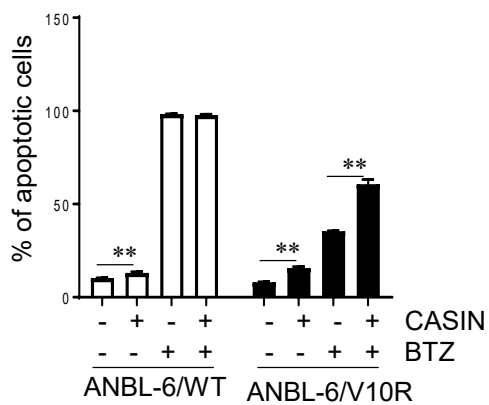

Supplement: Supplemental Figure S1 — Half-maximal growth inhibitory concentration (GI50) of CASIN in melphalan- and bortezomib-resistant MM cells. LR5 (A) and V10R (B) cells were plated at 10,000 cells/well and treated with or without different concentrations of CASIN for 2 days. Viable cells were measured using MTS as described in the Materials and Methods. [file Data_Sheet_1.pdf]
